# Upright or inverted, entire or exploded: right-hemispheric superiority in face recognition withstands multiple spatial manipulations

**DOI:** 10.7717/peerj.1456

**Published:** 2015-12-01

**Authors:** Giulia Prete, Daniele Marzoli, Luca Tommasi

**Affiliations:** Department of Psychological Science, Health and Territory, University of Chieti-Pescara, Chieti, Italy

**Keywords:** Face matching, Exploded faces, Face-inversion effect, Divided visual field paradigm, Hemispheric asymmetries

## Abstract

**Background.** The ability to identify faces has been interpreted as a cerebral specialization based on the evolutionary importance of these social stimuli, and a number of studies have shown that this function is mainly lateralized in the right hemisphere. The aim of this study was to assess the right-hemispheric specialization in face recognition in unfamiliar circumstances.

**Methods.** Using a divided visual field paradigm, we investigated hemispheric asymmetries in the matching of two subsequent faces, using two types of transformation hindering identity recognition, namely upside-down rotation and spatial “explosion” (female and male faces were fractured into parts so that their mutual spatial relations were left intact), as well as their combination.

**Results.** We confirmed the right-hemispheric superiority in face processing. Moreover, we found a decrease of the identity recognition for more extreme “levels of explosion” and for faces presented upside-down (either as sample or target stimuli) than for faces presented upright, as well as an advantage in the matching of female compared to male faces.

**Discussion.** We conclude that the right-hemispheric superiority for face processing is not an epiphenomenon of our expertise, because we are not often exposed to inverted and “exploded” faces, but rather a robust hemispheric lateralization. We speculate that these results could be attributable to the prevalence of right-handedness in humans and/or to early biases in social interactions.

## Introduction

The ability to recognize conspecifics is crucial for survival, and it has been demonstrated in a variety of species that visual cues are very important to this purpose, especially for social animals (i.e., Gronenberg, Ash & Tibbetts, 2007; Parr, 2011; Dahl et al., 2013; Somppi et al., 2014). One of the most useful visual cues for recognizing conspecifics is the face: a great amount of evidence has been collected on the ability to identify faces (e.g., Ramon, 2015), also indicating that the tendency to gaze at faces rather than other objects is an innate skill, not only in humans but also in other animals (Johnson et al., 1991; Sugita, 2008; Rosa Salva, Regolin & Vallortigara, 2010; Rosa Salva et al., 2011). Proof of the importance of facial recognition is the evidence of specific cerebral areas involved in face processing. A number of studies have shown that the fusiform gyrus, particularly in the right hemisphere, is the brain area specifically involved in face processing (Gross, Rocha-Miranda & Bender, 1972; Bruce, Desimone & Gross, 1981; McCarthy et al., 1997), so that this area has been named “fusiform face area” (Kanwisher, McDermott & Chun, 1997; Kanwisher & Yovel, 2006). Neuroscientists agree in identifying the right hemisphere as mostly involved in face processing (i.e., Rizzolatti, Umiltà & Berlucchi, 1971; Yovel, in press), a right-hemispheric—but not left-hemispheric—lesion causing prosopagnosia, that is the inability to recognize the identity of faces (Barton et al., 2002).

Several studies investigated the possibility that the right-hemispheric advantage in face processing is really specific for this type of stimuli, as opposed to a specialization in processing all stimuli that demand acquired expertise. In other words, some authors have cast doubts on the real face-specific superiority of the right hemisphere, favoring the alternative view according to which the right hemisphere would be superior in the processing of familiar stimuli, faces being a sub-category of this class (e.g., Diamond & Carey, 1986; Gauthier & Tarr, 1997).

However, a compromise between these two hypotheses is that the right-hemispheric superiority in face processing could be considered as advantageous specifically because of the high frequency of exposure to this familiar class of stimuli: it is reasonable, in fact, to hypothesize that because we are continuously exposed to conspecific faces in everyday life, our brain has evolved a specific neuronal circuit in order to selectively process these frequent stimuli in terms of both rapidity and accuracy. Some evidence in this regard have shown that humans are more capable to recognize own-race and own-gender faces than other-race and other-gender faces (Wright & Sladden, 2003; Wiese, Kaufmann & Schweinberger, 2014). This evidence agree with the hypothesis of a specialized neural circuit evolved because of the high frequency of exposure to faces.

The “face inversion effect” (Yin, 1969) is referred to the impaired performance of observers who are asked to recognize upside-down faces. In this circumstance, it was shown that the right hemisphere maintained its superiority in face processing with respect to the left hemisphere, even if its performance worsened with respect to the processing of upright faces. Moreover, the fusiform face area has been shown to process faces presented both in upright and upside-down orientation (Kanwisher, Tong & Nakayama, 1998).

A biological advantage would have led to evolve specific neural circuits devoted to processing faces as special stimuli, both at the phylogenetic and at the ontogenetic level (Vallortigara, 2012), due to the crucial role of this category of stimuli for survival. The assumption of a “cerebral social predisposition” is confirmed by a fast, subcortical route devoted to the processing of face-like stimuli in humans (Johnson, 2005), as well as in other species (Rosa Salva, Mayer & Vallortigara, 2015). Starting from this premise, we could hypothesize that the right-hemispheric superiority might also turn out to be resistant to other kinds of manipulations besides face inversion (Leder et al., 2001), as already demonstrated by means of geometric distortions (Hole et al., 2002), face caricaturing (Schulz et al., 2012), and so on. In this regard, Pichler and colleagues (2012) investigated the role of first-order *versus* second-order structure of faces in adaptation mechanisms: they used the so-called “exploded faces,” created by dividing faces into parts which were spaced apart from each other preserving the whole facial configuration (thus destroying the second-order structure), and “scrambled faces,” in which the facial configuration was destroyed by swapping face parts with each other (thus destroying the first-order structure). The authors found that exploded upright and upside-down faces generated identity after-effects on intact upright test faces, whereas scrambled faces did not, concluding that the face-like configuration (first-order structure) is necessary to activate face representations, and that the second-order structure of faces was contained in both orientations. These results were somehow in contrast with the idea according to which inverted faces should be processed as a mere collection of local features rather than as a global percept. The classical view on hemispheric competence about global/local processing is that of a right-hemispheric dominance for global analysis and a left-hemispheric dominance for local analysis (see Karim & Kojima, 2010 for a review). By means of inverted exploded faces, however, Pichler and colleagues (2012) showed that these stimuli allowed to sidestep this distinction and to investigate face processing by considering both global and local aspects altogether. In fact, in exploded faces, the spatial relationships among parts remain unaltered (i.e., the eyes are presented above the nose) and the local features are always present (the details of each part of the image remain intact), differently, for example, from blurred or scrambled images. Another study in which exploded faces were used (Moscovitch & Moscovitch, 2000, who referred to these stimuli as “fractured faces”), showed a “super face-inversion effect”: upside-down fractured faces were recognized worse than both upright fractured and upside-down whole faces (see also Moscovitch, Winocur & Behrmann, 1997).

A right-hemispheric superiority in matching the identity of famous faces has been shown by Cooper and colleagues (2007), in a divided visual field paradigm, in which a prime face was centrally shown and then followed by a lateral target face. The authors found that the right hemisphere was superior to the left hemisphere in the matching of prime and target faces when the two stimuli were identical, whereas the authors did not find any lateralized bias when the stimuli were different images of the same face, or when they depicted two different familiar faces. Despite these behavioral results, electroencephalographic recordings showed a similar right temporal N250 modulation for the first two conditions (prime and target being the same image or different images of the same face). Cooper and colleagues concluded that a behavioral left visual field advantage (right-hemispheric superiority) in matching prime and target stimuli occurred only when they were the same image, but the event-related potentials revealed that the right hemisphere processed in the same way prime and target faces both when they were the same image and two different images of the same face (thus, confirming the ability to recognize a face, beyond a specific image).

In an attempt to link the possible right-hemispheric superiority in face recognition with the processing of configural aspects of face stimuli, we investigated hemispheric abilities in the processing of upright, inverted, entire and exploded faces (and the combinations of these conditions), by means of a divided visual field paradigm. The hypothesis of the study was that, starting from the well-established right-hemispheric dominance in face processing, the right hemisphere should appear dominant also in the processing of exploded faces, confirming the cerebral asymmetry also for faces undergoing different spatial manipulations. To investigate this issue, in a same/different forced choice task we presented a sample face in the center of the screen, followed by the presentation of a lateralized target face, exploiting the tachistoscopic unilateral visual field paradigm. By manipulating the spatial orientation (upright/inverted) and the global configuration (different degrees of explosion) of the stimuli, we aimed to confirm that the right-hemispheric superiority in face processing would withstand to multiple spatial manipulations.

## Materials and Methods

### Participants

Thirty-seven volunteers (32 females) took part in the experiment (mean age: 24.73 ± 0.29 years). As in previous studies investigating the effects of face perception, the gender of participants was not controlled for (e.g., Ramon & Rossion, 2012; Cattaneo et al., 2014; Bourne & Hole, 2006), starting from the evidence that females and males did not differ in recognizing lateralized presented faces (Hirnstein, Hausmann & Güntürkün, 2008; Godard & Fiori, 2010). All participants had normal or corrected-to-normal vision and were naïve about the purpose of the experiment. Two participants were left-handed and the other 35 were right-handed, as self-reported (left-handed participants were not excluded from the sample, as in previous studies in which lateralized faces were used as stimuli, e.g., Ramon & Rossion, 2012). The present study did not involve patients, children or animals, as well as drugs, genetic samples or invasive techniques, thus it was not subject to ethical review by the academic medical research board. Nevertheless, informed consent was obtained from all participants and the experiment was conducted in accordance with the ethical standards prescribed by the Declaration of Helsinki.

### Stimuli

**Figure 1:**
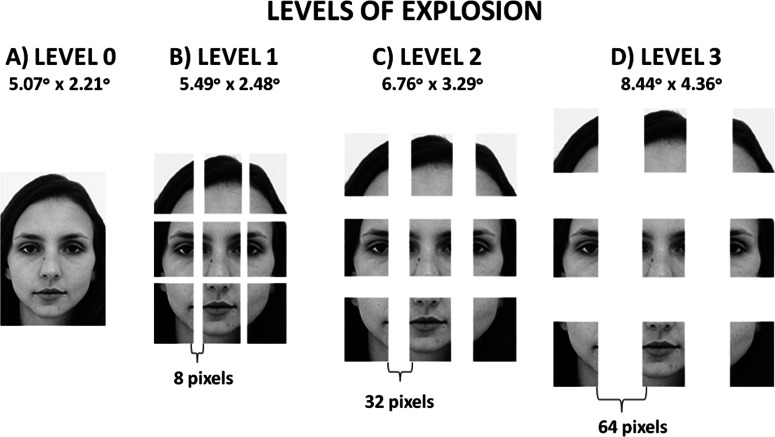
Example of Stimuli preparation. Example of a stimulus at each level of explosion created for illustrative purpose (these images are not included in the database used in the study): (A) level 0 (the face is shown in its entirety); (B) level 1 (the parts of the image are spaced 8 pixels from one another); (C) level 2 (the parts of the image are spaced 32 pixels from one another); (D) level 3 (the parts of the image are spaced 64 pixels from one another).

Stimuli were created from photographs contained in the Karolinska Directed Emotional Faces (Lundqvist, Flykt & Öhman, 1998), a database of female and male faces depicted in emotional and neutral poses. Photographs of 3 female and 3 male Caucasian actors in neutral pose and in frontal view were selected and rendered in gray scale. In order to make the gender of faces well-distinguishable, all female actresses had long hair and all male actors had short hair. From each original photograph, a total of 4 images were obtained by means of the software Photoshop (Adobe Systems Inc., San Jose, CA, USA). Specifically, in order to create the “exploded faces,” 3 more images were created based on each original photograph, by cutting the original image into 9 parts having the same size, according to a virtual 3 × 3 grid. From the original image measuring 192 × 132 pixels (height × width, visual angle: 5.07° × 2.21°, seen at a distance of 72 cm), 9 parts were thus obtained, each measuring 64 × 44 pixels (1.69 × 0.74°). In order to create the exploded stimuli, the 8 external parts in the image were displaced away from the central part. The distance of the external parts from the central one determined the “level of explosion”: at level 0, the face was presented in its entirety—since all the pieces were juxtaposed to each other (original photograph); at level 1, each part was orthogonally spaced 8 pixels away from the neighboring parts; at level 2, each part was orthogonally spaced 32 pixels away from the neighboring parts; at level 3, each part was orthogonally spaced 64 pixels away from the neighboring parts (distances are intended from side to side, see Fig. 1). Stimuli were first created in canonical orientation (upright orientation), and a second set was then obtained rotating each stimulus 180° (inverted orientation).

### Procedure

The task was controlled by means of E-Prime software (Psychology Software Tools Inc., Pittsburgh, PA, USA) and presented on a screen with a resolution of 1,280 × 1,024 pixels. Participants were tested individually, in a darkened room, sitting at a distance of 72 cm from the computer screen.

**Figure 2:**
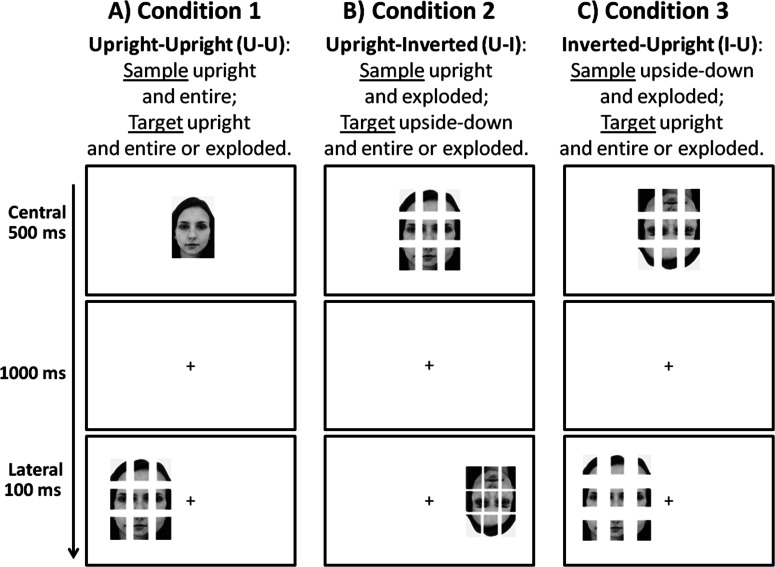
Schematic representation of the procedure. Schematic representation of the paradigm: a sample face was centrally presented for 500 ms, followed by a fixation cross lasting 1,000 ms, then a target face was presented for 100 ms in either the left visual field (LVF) or in the right visual field (RVF). (A) Condition 1 (sample: upright and entire; target: upright and entire or exploded); (B) Condition 2 (sample: upright and exploded; target: upside-down and entire or exploded); (C) Condition 3 (sample: upside-down and exploded; target: upright and entire or exploded).

Participants were randomly assigned to one of three experimental conditions, which had a similar structure, but differed for the spatial manipulations of sample and target stimuli (see Fig. 2). In all conditions the level of explosion of the sample face was blocked among trials, whereas the target face was presented at each of the four levels of explosion.

A trial started with a sample face shown for 500 ms in the center of the screen; then a fixation cross was centrally presented for 1000 ms, and in the following 100 ms a target face was presented on the left side of the screen (50% of trials) or on the right side of the screen (50% of trials), the central fixation cross remaining visible (the center of the stimuli was placed at a distance of 5.37° of visual angle to the left or to the right of the central fixation cross). Then the screen became blank until the participant gave her/his response and—1,000 ms after the response—the next trial started. Target stimuli were presented for 100 ms in order to reduce the possibility of eye movements, starting from the evidence that not less than 110–120 ms are needed to carry out a saccadic movement (i.e., Trottier & Pratt, 2005).

There were 32 trials for each of the 6 identities (3 female and 3 male): in 16 of such trials, the target face was the same as the sample face (i.e., female 1), in the other 16 trials the identity of the target face was different with respect to the identity of the sample face (i.e., in 8 of the unmatched trials in which the sample face was female 1, the target face was female 2, and in the other 8 trials the target face was female 3). The resulting 192 trials in each experimental condition were repeated three times.

In the first experimental condition the sample face was presented upright and entire (level of explosion 0), and the target face was presented upright and at each of the 4 levels of explosion (levels: 0, 1, 2 and 3). This condition, in which sample and target stimuli are presented upright (from now on condition “Upright–Upright”), was administrated to 11 participants (7 females). In the second condition a further element of spatial manipulation was added, presenting the stimuli upside-down, as well as exploded: sample faces were presented upright and at level of explosion 2, and target faces were presented upside-down and at each of the 4 levels of explosion. This condition, in which the sample face was presented upright and the target face was presented in inverted spatial orientation (from now on condition “Upright–Inverted”), was administrated to 13 participants (12 females). In the third condition, the spatial orientation of the sample stimuli was also manipulated, in order to investigate the possibility to alter the expected right-hemispheric superiority when the initial coding was more difficult, due to face inversion: sample faces were presented upside-down and at level of explosion 2, and target faces were presented upright and at each of the 4 levels of explosion. This condition, in which the sample face was presented in inverted spatial orientation and the target face was presented in canonical spatial orientation (from now on condition “Inverted-Upright”), was administrated to 13 participants (all females).

Written instructions were presented at the beginning of the task, in which participants were required to maintain their gaze on the central fixation cross and half of them were asked to press either a keyboard button with the left hand when the identity of the target face was the same as that of the sample face, or a button with the right hand when the identity of the sample and target faces differed, whereas the other half received the instructions with the opposite association between hand and response. Prior to the beginning of the task, participants were informed that stimuli could be presented as upright or upside-down, entire or exploded and they were shown some examples in order to familiarize with the stimuli. They were also informed that the gender of faces remained the same between sample and target stimuli in each trial.

The presentation order of the trials was randomized within and across participants, and the task lasted about 25 minutes.

## Results

Results were analyzed by means of the Statistica 8.0.550 software (StatSoft. Inc., Tulsa, OK, USA). In a first step, the frequencies of correct responses of the whole sample were transformed in percentages and the mean was compared to the 50% chance threshold by means of exact *t*-tests. This first analysis showed that participants did not respond by chance, since the *t*-test was significant (*t*_(36)=_12.79; *p* < .001), indicating that participants matched the identity of sample and target faces at a higher rate than chance (mean accuracy ± SEM: 61.71% ± 0.91%). The results were significant also considering each of the 3 groups separately (Upright–Upright: *t*_(10)_ = 17.92; *p* < .001; Upright–Inverted: *t*_(12)_ = 5.73; *p* < .001; Inverted-Upright: *t*_(12)_ = 7.6; *p* < .001).

A split-plot analysis of variance (ANOVA) was carried out, using Sex of faces (female, male), Hemifield of presentation of the target face (left visual field: LVF, right visual field: RVF) and Level of explosion of the target face (0, 1, 2, 3) as within-subject factors, Condition (Upright–Upright, Upright–Inverted, Inverted-Upright) as between-subject factor, and the percentage of correct responses as the dependent variable. Since we did not have specific hypotheses concerning the effect of gender and handedness of participants, these factors were not controlled for, and thus they were not included in the analysis. Post-hoc comparisons were computed by means of Duncan tests.

All main effects were significant. The effect of Hemifield (*F*_(1,34)_ = 99.99, *p* < .001, ${\eta }_{p}^{2}=.746$), revealed that the target faces were correctly recognized more frequently when presented in the LVF (66.32 ± 0.67) than in the RVF (57.10 ± 0.51). The main effect of Sex of faces (*F*_(1,34)_ = 91.61, *p* < .001, ${\eta }_{p}^{2}=.729$) showed that Female faces were correctly recognized more frequently than Male faces (female faces: 65.73 ± 0.67; male faces: 57.68 ± 0.55). Post-hoc comparisons on the main effect of the Level of explosion (*F*_(3,102)_ = 10.65, *p* < .001, ${\eta }_{p}^{2}=.238$; level 0: 67.14 ± 0.94; level 1: 63.00 ± 0.97; level 2: 61.20 ± 0.90; level 3: 59.49 ± 0.87) showed that the correct matching rates were higher when target faces were presented at Level of explosion 0 with respect to Levels of explosion 2 (*p* = .017) and 3 (*p* < .001), and at Level of explosion 1 with respect to Levels of explosion 2 (*p* = .020) and 3 (*p* < .001). There were no differences between Levels 0 and 1, and between Levels 2 and 3. Finally, also the effect of Condition was significant (*F*_(2,34)_ = 7.43, *p* = .002, ${\eta }_{p}^{2}=.304$), and post-hoc comparisons showed that target faces were correctly recognized more frequently in Upright–Upright condition (65.89 ± 0.72) than in Upright–Inverted (58.34 ± 0.76; *p* < .001) and in Inverted-Upright (61.53 ± 0.82; *p* = .031) conditions.

The significant interaction between Hemifield and Sex of faces (*F*_(1,34)_ = 28.82, *p* < .001, ${\eta }_{p}^{2}=.459$) confirmed that Female faces were correctly recognized more frequently than Male faces in both the LVF and the RVF, and that both Female and Male faces were correctly recognized more frequently when presented in the LVF than in the RVF (*p* < .001 for all comparisons).

**Figure 3:**
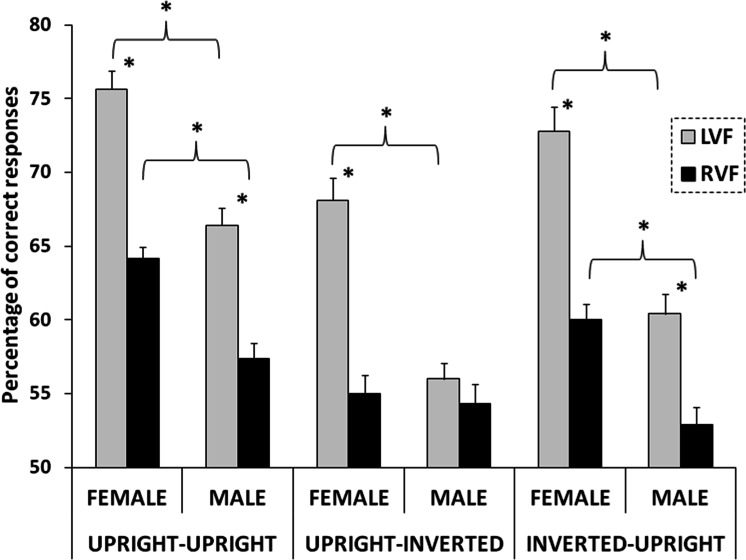
Results. Interaction Hemifield X Sex of face X Condition: percentage of correct recognition of female and male target faces presented in the left visual field (LVF, gray columns) and in the right visual field (RVF, black columns), in Upright–Upright, Upright–Inverted and Inverted–Upright conditions. Error bars represent standard errors.

Finally, also the three-way interaction concerning Hemifield, Sex of faces and Condition was significant (*F*_(2,34)_ = 5.01, *p* = .012, ${\eta }_{p}^{2}=.228$; Fig. 3). Post-hoc comparisons showed a LVF advantage in all cases (*p* < .001 for all comparisons between LVF and RVF), with the exception of male faces in Upright–Inverted condition. Moreover, female faces were correctly recognized more frequently than male faces in both visual fields and in any condition (*p* < .001 for all comparisons), with the exception of faces presented in the RVF in the Upright–Inverted condition.

## Discussion

The present study confirms the right-hemispheric superiority in face processing, extending this evidence to the unusual situation constituted by exploded and inverted faces. Indeed, the effect of the visual field was highly significant, showing that the identity of two faces was better matched when target stimuli were presented in the left visual field (right hemisphere) than in the right visual field (left hemisphere). This evidence is in line with a number of studies (Bourne & Hole, 2006; Cattaneo et al., 2014), strengthening the hypothesis of a right-hemispheric superiority in face processing, that goes beyond the idea of expertise with a specific class of stimuli: in fact, if this latter case can be applicable to the identification of an upright and entire face—a class of stimuli to which we are continuously exposed—it is not applicable to exploded and/or inverted faces. The results of the present study are also in line with those of Cooper and colleagues (2007), who found a similar right-hemispheric N250 modulation during the recognition of a target face that followed a prime face of the same identity, both when prime and target were the same image and when they were different images (even if the authors lacked to find a behavioral LVF advantage when prime and target were different images of the same person). These results showed that the right-hemispheric superiority in face recognition is not just based on a match-to-sample processing due to a pure mnemonic encoding of the perceptual characteristics of the stimuli, but it is based on higher-order processes, which seem to be resistant to perceptual changes (spatial manipulations or different views of the face). In line with this view, in a facial identity adaptation paradigm during fMRI, Verosky & Turk-Browne (2012) found that adaptation mechanisms occurred in the left fusiform face area only when a face had previously been processed by the RH, whereas they did not occur when the face had only been processed by the LH. The authors concluded that facial identity information is mainly processed by the RH, and then it can be transferred from the right to the left hemisphere. As regard the spatial manipulation of the stimuli, in a PET study Rossion and colleagues (2000) showed that cerebral asymmetries for part-based *versus* whole-based processing of faces take place in the fusiform gyrus, more activated in the LH for part-based processing, and in the RH for whole-based processing.

The present study also showed that exploded faces were recognized with more difficulty than entire faces, and specifically that the entire or almost entire stimuli (levels of explosion 0 and 1) were better recognized than the exploded stimuli (levels of explosion 2 and 3), independently of their spatial orientation (i.e., upright/upside-down). We also found that the performance of the participants were lower when either the sample or the target face were presented exploded and in inverted orientation (Conditions 2 and 3) than when they were presented exploded and upright (Condition 1), in accordance with the “super face-inversion effect” (Moscovitch & Moscovitch, 2000). The results of the present study showed that female faces were better recognized than male faces. Importantly, however, as regard the hemispheric bias in interaction with the gender of the stimuli, the only occurrence in which the right-hemispheric superiority was not evident was that concerning male faces in condition “upright-inverted,” in which after the upright sample face, an upside-down target face was presented.

An alternative explanation for the right-hemispheric superiority in face analysis is linked to a leftward spatial bias possibly attributable to the prevalence of right-handedness in humans and the higher likelihood of scanning the left side of others in face-to-face interactions (for a detailed review, see Marzoli, Prete & Tommasi, 2014). Moreover, a further explanation for the right-hemispheric superiority in face recognition, and in particular for female faces, takes into account an additional factor, concerning the early interactions between infants and caregivers, mainly mothers, namely a leftward bias in maternal cradling, that could be attributable to the fact that in this way the infant’s facial signals directly reach the mother’s right hemisphere, preferentially devoted to face processing (i.e., Huggenberger et al., 2009; see also Marzoli, Prete & Tommasi, 2014) and to emotion recognition (Gainotti, 1972; Prete et al., 2015a; Prete et al., 2015b). In line with this idea, it could be hypothesized that female faces are better recognized than male faces when presented in the LVF (right hemisphere) and in inverted orientation (“Upright–Inverted” condition), because of the exposure to the upside-down perspective of a female face in the early childhood: in this period, in fact, infants are held by mothers (preferentially on their left side) and are more likely exposed to the mother’s face from a variety of vantage points. Accordingly, a right-hemispheric bias for the recognition of female faces, using chimeric faces, was also found in both female and male observers by means of a gender recognition task carried out by Parente & Tommasi (2008), supporting the right-hemispheric dominance in female-face processing, independently of the observers’ gender. Moreover, no difference in the accuracy between female and male observers were found in a divided visual field paradigm in which participants were required to discriminate faces from non-faces (Hirnstein, Hausmann & Güntürkün, 2008), or to recognize the identity of a face (Godard & Fiori, 2010).

To conclude, the present study confirms the right-hemispheric superiority in face processing and extends this ability also to stimuli transformed by multiple spatial manipulations (inverted and exploded faces). We speculate that this hemispheric superiority could possibly be linked to both right-handedness and the leftward bias in maternal cradling (Marzoli, Prete & Tommasi, 2014). This circumstance casts doubts on the possibility of a right-hemispheric superiority for all categories of stimuli with which we have expertise, because it is evident that exploded faces are unusual stimuli for observers (even more so if they are also inverted). The present findings should be intended as a further confirmation of the right-hemispheric dominance in face processing, showing the robustness of this neuropsychological fact even in cases of extreme spatial manipulations of the stimuli. These conclusions need further investigations. For instance, it has to be noticed that in the present study participants were asked to maintain their initial position during the whole task and to fixate the central cross, but neither eye movements nor head position were monitored, thus caution is needed in the interpretation of the present results, and possible further studies should investigate the strength of the present conclusions by adding direct measurements of both eye movements and head position during the lateralized presentation of the stimuli. Moreover, the hypothesis drawn in this study could be tested by using stimuli other than faces in order to directly compare performance and hemispheric abilities in the matching of different classes of stimuli, as well as by comparing the performance of female and male participants.

## Supplemental Information

10.7717/peerj.1456/supp-1 Supplemental Information 1 Raw data Raw data and supplementary analyses.

## Additional Information and Declarations

### Data Availability

The following information was supplied regarding data availability: The raw data can be found in Supplemental Information 1.
